# Coronary Artery Anomalies in Common Arterial Trunk: Proposal of a New Anatomical Classification

**DOI:** 10.1093/icvts/ivag083

**Published:** 2026-03-21

**Authors:** Edouard Long, Minji Ho, Vitaliy Androshchuk

**Affiliations:** Faculty of Life Sciences and Medicine, King’s College London, London SE1 1UL, United Kingdom; Institute of Cardiovascular Science, University College London, London WC1E 6DD, United Kingdom; Faculty of Life Sciences and Medicine, King’s College London, London SE1 1UL, United Kingdom; Faculty of Life Sciences and Medicine, King’s College London, London SE1 1UL, United Kingdom

**Keywords:** common arterial trunk, truncus arteriosus, coronary artery anomalies, cardiac surgery, congenital heart disease

## Abstract

Coronary artery abnormalities (CAAs) are frequently encountered in common arterial trunk (CAT), with an estimated incidence of 5%-20%. However, their prognostic implications remain unclear. Surgical challenges potentially arise due to coronary arteries crossing the right ventricular outflow tract (RVOT), close proximity of the coronary and pulmonary orifices, and distortion to the proximal coronary segments and ostia during arch reconstruction or truncal valve replacement. Some studies have demonstrated that CAAs confer worse outcomes after CAT repair, while others have reported no significant prognostic impact. Both the number and subtype of CAAs may influence outcomes, but heterogeneous categorization limits the conclusions that can be drawn from existing studies. A uniform classification of CAAs in CAT is warranted to better ascertain the prognostic impact of CAA burden and morphology. This may enable more focused decision-making in clinical scenarios where a high-risk CAA pattern is suspected. For example, it may help inform the intraoperative trade-off between probing the coronaries to define their precise morphology against the risk of causing damage. We propose a classification consisting of 6 abnormalities: (1) single coronary artery, (2) ostial stenosis, (3) intramural course, (4) juxtacommissural origin, (5) coronary crossing RVOT, and (6) close proximity of coronary and pulmonary orifices.

Coronary artery abnormalities (CAAs), congenital lesions characterized by abnormal origin or course of the epicardial coronary arteries, are associated with common arterial trunk (CAT), also known as truncus arteriosus. They are common in CAT patients, with an estimated incidence of 5%-20%, but their specific morphology and prognostic implications remain unclear.^1^^,^^2^ Although pathological and clinical studies have investigated the relationship between CAT and CAAs, a definitive consensus on the classification of CAAs in CAT is lacking. This is likely due to the heterogeneous patterns in coronary anatomy, complicating efforts to discern the prognosis of CAAs in CAT patients.

The CAA most often reported in CAT is a single coronary artery (SCA), in which a solitary coronary vessel arises from a single coronary ostium, with substantial heterogeneity noted in its origin and course.^1–3^ Other CAAs include ostial stenosis, juxtacommissural origin, and intramural courses.^1^ The location of the left and right coronary ostia can vary in CAT, although they are predominantly located below the sinotubular junction within their respective sinuses of Valsalva.^4^^,^^5^ Of relevance is the proximity of the pulmonary and coronary orifices, as this may affect the risk of injury to nearby coronaries during excision of the pulmonary artery and closure of the resulting defect.^4^^,^^5^ In a dedicated study of ex vivo heart specimens, Adachi et al^4^ identified “close proximity,” defined as ≤2 mm, between the coronary and pulmonary orifices in 13/56 (23%) of their specimens, which largely affected the left coronary artery (12/13 cases) and was associated with a sinusoidal pulmonary origin. Another important consideration is the course of the coronary arteries, as those that cross the right ventricular outflow tract (RVOT) are at greater risk of damage during a right ventriculotomy or from compression by an overlying conduit.^6^^,^^7^ It should be noted that “close proximity” and/or coronary artery course crossing the RVOT, in the absence of an intrinsic coronary lesion, may not necessarily be considered as a CAA. However, in the context of CAT, it clearly adds significant operative complexity.

CAAs may pose additional technical challenges and complicate CAT operations. In cases involving reconstruction of an interrupted aortic arch or truncal valve replacement, the risk of distortion to the proximal coronary segments and ostia is increased, which patients with CAAs may be less able to tolerate.^8^ For example, paediatric patients are unlikely to have collateral coronary circulations, as they usually do not have coronary artery disease. Therefore, any degree of impedance to coronary flow may inevitably lead to infarction.^7^ Intramural CAAs may be particularly susceptible due to the presence of an ostial ridge, compression of the intramural segment, or kinking secondary to the acute take-off angle.^9^ Excision of the pulmonary arteries from the truncal root may jeopardize the coronary arteries, particularly when a high left coronary ostium is located immediately beneath the origin of the pulmonary trunk. Additionally, the coronary arteries are at risk during ventriculotomy and may be compromised by sutures placed to secure a conduit during neoaorta reconstruction. Furthermore, homograft-related compression of an anomalous coronary artery may also result in myocardial ischaemia. These risks may be amplified when the coronary arteries cannot be adequately visualized intraoperatively due to pericardial fibrosis from prior surgical intervention, underscoring the importance of thorough pre-operative imaging and documentation. Although not always routinely performed during the initial intervention in CAT, CAAs can be surgically corrected using techniques including osteoplasty, coronary unroofing, and reimplantation.^1^

Evidence suggests that CAAs may confer a worse prognosis in CAT patients. This was recently demonstrated by Bonilla-Ramirez et al,^1^ who studied the prognostic impact of CAAs in a single-centre cohort of 107 patients from 1995 to 2019, with a median follow-up of 7 years. The authors categorized patients by the number of lesions and found that the presence of CAAs, apart from SCA, was associated with increased mortality, with a clear delineation between the number of CAAs and mortality. CAAs have also been identified as a risk factor for adverse outcomes by several other authors,^2^^,^^10^ although some have reported conflicting results. This includes a cohort study by Urban et al,^6^ in which all 16 (35%) patients with CAAs were alive at the end of the 10-year follow-up period. Aside from small sample sizes (raising the chance of type II error), differences in surgical technique between centres, variations in CAT type and other clinical characteristics, inconsistent definitions of CAAs, and the assumption that all CAAs carry the same prognosis complicate efforts to determine their true impact. This is particularly important because SCA and the number of CAAs may confer different levels of risk within individual patients. Naimo et al^2^ demonstrated the prognostic significance of having a major coronary artery crossing the RVOT in a cohort of 171 CAT patients, reporting that 3/4 (75%) patients displaying this anomaly had died, at 4 days, 77 days, and 17.5 years post-CAT repair, respectively. However, this finding was not replicated by Bonilla-Ramirez et al. This discrepancy may be explained by the fact that the affected patients in the Naimo et al cohort underwent surgery decades earlier before subsequent improvements in surgical techniques.

As the significance of CAAs in CAT is difficult to interpret due to heterogeneous reporting, consistent categorization of these CAAs is needed to enable more robust conclusions about their clinical impact and to better inform their management. Since multiple CAAs may coexist within the same coronary system, for example, intramural coronaries frequently have ostial stenosis due to angulation, it is important to document both the number and type of individual CAAs to quantify the overall lesion burden. From the existing literature, it is challenging to ascertain whether the number and/or type of CAA are prognostically relevant. Therefore, consistent classification based on pre-intervention cross-sectional imaging or intraoperative inspection would be helpful, as it would enable future systematic retrospective assessment of prognostic implications. As the significance of CAA morphology and burden becomes clearer, it may enable more focused investigation in situations where a “high-risk” CAA pattern is suspected. This may help inform the intraoperative trade-off between thoroughly probing the coronaries to define their precise morphology and performing surgical correlation, which carries the potential risk of causing damage.

The proposed classification of CAAs in CAT for consideration is illustrated in **Figure 1**. Six abnormalities are defined which can exist in isolation or in tandem within a coronary system: (1) single coronary artery—a single coronary vessel arising from a single coronary ostium, (2) ostial stenosis—a narrowing and/or blockage of the coronary ostium, (3) intramural course—a coronary vessel which runs within the wall of the arterial trunk, (4) juxtacommissural origin—a coronary vessel which arises at/near the commissure of the truncal valve, (5) coronary crossing the RVOT—a coronary vessel which crosses the RVOT, and (6) close proximity of coronary and pulmonary orifices—coronary and pulmonary orifices lie within close proximity to each other (∼≤2 mm). As with all congenital lesions, there may exist exceedingly rare CAA variants that do not fit into these exact categories, but the existence of these must be balanced against the need for a pragmatic and streamlined classification. Moreover, variants in truncal valve anatomy, such as bicuspid and quadricuspid configurations, should be documented alongside the morphology of the valvular sinuses and coronary ostia. In keeping with prior descriptions, individual leaflets are designated according to their topographical location within the antero-posterior and left-right planes.^7^ Leaflet dysplasia may also be present and should be noted.

**Figure 1.**
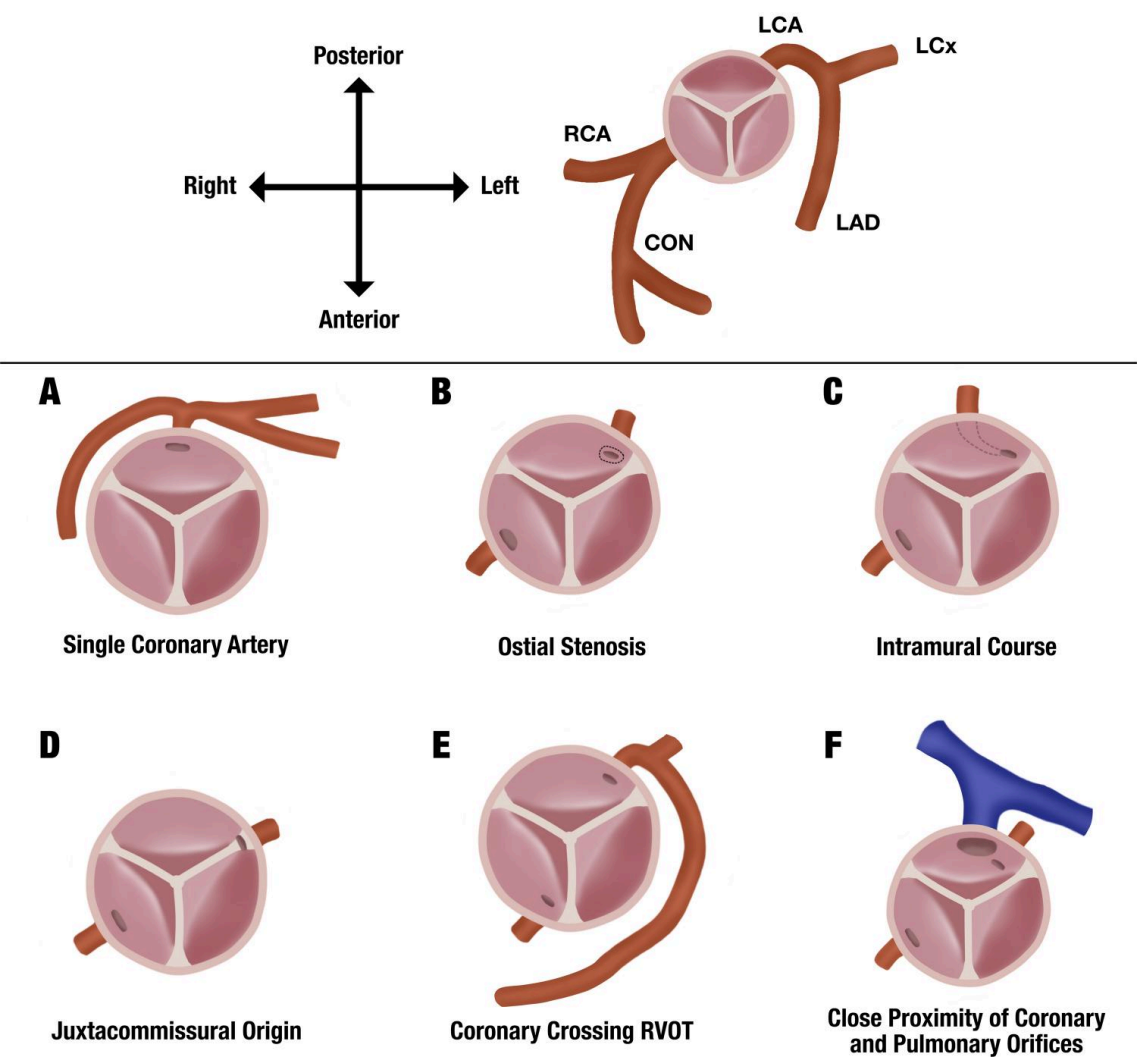
Classification of Coronary Artery Abnormalities in Common Arterial Trunk. Top panel shows representative coronary anatomy in CAT without any CAAs. Bottom panel outlines the proposed 6 part CAA classification: (A) single coronary artery—a single coronary vessel arising from a single coronary ostium, (B) ostial stenosis—a narrowing and/or blockage of the coronary ostium, (C) intramural course—a coronary vessel which runs within the wall of the arterial trunk, (D) juxtacommissural origin—a coronary vessel which arises at/near the commissure of the truncal valve, (E) coronary crossing the RVOT—a coronary vessel which crosses the RVOT, and (F) close proximity of coronary and pulmonary orifices—coronary and pulmonary orifices lie within close proximity to each other (∼≤2 mm). Abbreviations: CAA, common arterial trunk; CAT, common arterial trunk; CON, conal artery; LAD, left anterior descending artery; LCA, left coronary artery; LCx, left circumflex artery; RCA, right coronary artery; RVOT, right ventricular outflow tract.

In conclusion, CAAs are frequent in CAT patients, most often presenting as SCA. CAAs complicate surgery and may impact prognosis, though their significance remains unclear. Both the number and subtype of CAA may potentially influence outcomes, but heterogeneous categorization limits the conclusions that can be drawn from existing studies. A standardized classification of CAAs in CAT, such as the one outlined in this manuscript, is warranted to better determine the prognosis of CAA burden and morphology.

## Data Availability

The data underlying this article are available in the article.
